# Synthesis of Linear and Cyclic Disulfide Heptapeptides of Longicalycinin A and Evaluation of Toxicity on Cancerous Cells HepG2 and HT-29

**Published:** 2018

**Authors:** Mohammad Hassan HoushdarTehrani, Abdolhamid Bamoniri, Bi Bi Fatemeh Mirjalili, Mohammadreza Gholibeikian

**Affiliations:** a *Department of Pharmaceutical Chemistry, School of Pharmacy, Shahid Beheshti University of Medical Sciences, Tehran, Iran. *; b *Protein Technology Research Center, Shahid Beheshti University of Medical Sciences, Tehran, Iran. *; c *Department of Organic Chemistry, Faculty of Chemistry, University of Kashan, Kashan, Iran. *; d *Department of Chemistry, College of sciences, Yazd University, Yazd, Iran.*

**Keywords:** Longicalycinin A analogue, Disulfide band, Anticancer, MTT assay, Apoptosis

## Abstract

In this work, linear and cyclic disulfide heptapeptides of Longicalycinin A have been successfully synthesized by solid phase methodology with Fmoc /t-Bu and solution phase, respectively. 2-Chlorotrityl chloride resin (2-CTC) was used as a solid support. The synthesized linear disulfide analogue of Longicalycinin A was cleaved from the resin as a protected peptide. The final deprotection was performed by treatment with TFA 95% containing scavengers to achieve the deprotected linear disulfide analogue of Longicalycinin A which was characterized by different instrumental methods using LC-MS and FT-IR. Macrocyclization of deprotected linear peptide was done by an oxidating reagent. Linear and cyclic disulfide heptapeptides of Longicalycinin A were evaluated their toxic activity against cell lines of HepG2 and HT-29 using 3- (4, 5-dimethylthiazol-2-yl) -2, ­5-diphenyltetrazolium bromide reagent in MTT assay. The synthetic analogues showed a relative good activity against cell lines of HepG2 and HT-29 with IC_50_ values from 10.33 µg/mL to 12.45 µg/mL, in comparison to the standard drug 5-fluorouracil (5-FU). Safety profiles of the synthesized linear and cyclic disulfide analogues of Longicalycinin A were also examined on skin fibroblast cells. Between the linear and cyclic disulfide heptapeptides of Longicalycinin A, the cyclic peptide showed a considerable toxic activity on the cancerous cell lines along with a low safety result on normal cells. Therefore, the linear disulfide heptapeptide of Longicalycinin A would be encouraging to develop new anticancer agents.

## Introduction

The formation of disulfide bridges is often a crucial final stage in peptide synthesis. Disulfides in proteins play an important role in the maintenance of biological activity and conformational stability (1-5). There are compelling evidences that the disulfide pattern can be critical in the folding of proteins by decreasing entropy and providing a favorable local interaction, as it is a common strategy to design bis-cysteine cyclic peptides (6, 7). In chemistry a disulfide refers to a functional group with the general structure R-S-S-Rʹ. The linkage is also called an SS bond or a disulfide bridge and is usually derived by the coupling of two thiol groups. A disulfide bond is formed when a sulfur atom from one cysteine forms a single covalent bond with another sulfur atom from a second cysteine residue located in a different part of the proteins. These bonds help stabilize proteins, particularly those secreted from cells. The formation of disulfide bonds requires the proper management of cysteine residues, including first protecting and then later removing side groups and properly pairing the cysteine residues (8). Head-to-tail disulfide bonds are integral components of the three-dimensional structure of many proteins (9)­. These covalent bonds are found in almost all classes of extracellular peptides and proteins. The straightest approach for the preparation of disulfide-containing peptides involves initial assembly of the linear chain, using the same (invariable) acid-labile protecting group for all cysteine residues. The structures of some such protecting groups, compatible with solid-phase peptide synthesis (SPPS) methods, are:* S*-Tmob, *S*-Mmt (10), or *S*-Trt (11) for Fmoc SPPS, and *S*-Mob (12) or *S*-Meb (13) for Boc SPPS. The resultant precursor, in which all cysteine residues should be in the free sulfhydryl form, can then be subjected in an aqueous solution by a mild oxidization, keeping in mind that the precise experimental conditions, e.g., pH, ionic strength, organic cosolvent, temperature, time, and concentration, can often make a substantial difference in terms of the quality of disulfide product obtained (14, 15). High dilution is recommended to minimize physical aggregation, and also to minimize the chemical formation of dimers, oligomers, or intractable polymers. An oxidizing agent is used for the disulfide formation. The optimal pH range for disulfide formation is between 3 and 8. The protein denaturation may occur if the pH is lower than 3. The disulfide interchanging is too fast at pH higher than 8. Oshima *et al*. isolated several dianosides from the plant *Dianthus superbus var. Longicalycinus.* This plant has been used as a diuretic, anti-inflammatory, analgesic agent and examined for hepatotoxic activity (16-19)­ Further studies on this plant resulted in the isolation of Longicalycinin A, [cyclo-(Gly^1^-Phe^2^-Pro^3^-Tyr^4^-Phe^5^)], a new cytotoxic cyclic peptide by Hsieh *et al* (20). Recent work has been focused on the synthesis of Longicalycinin A (21). The aim of this study was to design and synthesize linear and cyclic disulfide heptapeptides of longicalycinin A that might be expected to produce anticancer activity more than the original peptide. In this work, the designed peptides were synthesized *via* solid phase synthesis, and macrocyclization of the deprotected linear disulfide heptapeptide of Longicalycinin A was carried out by an oxidizing reagent in solution phase (22-23) at pH 8–9 (7, 15). The oxidation reaction was completed under gentle stirring in 8 hours. The cytotoxic activities of synthesized peptides were evaluated against various human cell lines including, HepG2 (human liver cancer cell line) and HT-29 (human colorectal adenocarcinoma cell line). In addition, the safety profile of these peptides was examined using human skin fibroblast cells. 

## Experimental

All commercially reagents and solvents were purchased from Merck, Aldrich and Fluka chemical companies and used without further purification. Flash column chromatography was carried out using silica Gel 60 (particle size 0.04–0.06 mm / 230–400 mesh). FTIR spectra were performed on Shimadzu recording and Nicolet Magna 550 spectrometers. The mass spectral measurements were performed on a 6410 Agilent LC-MS triple quadrupole mass spectrometer with an electrospray ionization (ESI) interface, USA. The cell lines were purchased from Iranian Biological Resource Center (IBRC), Tehran, Iran.

*The procedure for the synthesis of protected linear disulfide heptapeptide of Longicalycinin A: Cys(Trt)-Phe-Tyr­(tBu)-Pro-Phe-Gly Cys(Trt)*

Fmoc-Cys (Trt)-OH (1170 mg, 2 mmol) was attached to the 2-CTC resin with DIPEA (1mL) in anhydrous DCM: DMF (30 mL, 1:1) at room temperature for 2 h. After filtration, the remaining trityl chloride groups were capped by a solution of DCM / MeOH / DIPEA (17:2:1; 20 mL) for 30 min. Then, it was filtered and washed thoroughly with DCM (1 × 10 mL), DMF (2 × 20 mL). The resin-bound Fmoc-amino acid was treated with Piperazine 10% in DMF (100 mL) for 30 min and the resin was washed with DMF (4 × 20 mL). Then, a solution of Fmoc-Gly-OH (600 mg, 2.01 mmol), HATU (650 mg, 1.7 mmol), and DIPEA (0.5 mL) in 10 mL DCM was added to the resin-bound free amine and shaken for 2 h at room temperature. After completion of coupling, the resin was washed with DMF (2 × 10 mL), treated with Piperazine 10% in DMF (100 mL) for 30 min and washed again with DMF (4 × 20 mL). Other protected amino acids, i.e., Fmoc-Phe-OH­, Fmoc-Pro-OH, Fmoc-Tyr (tBu)-OH­, Fmoc-Phe-OH and Fmoc-Cys (Trt) - OH were added to the resin-peptide, sequentially, with the same procedure mentioned as above. During the synthesis, for detecting the presence or absence of free primary amino groups on the resin-peptide, chloranil test was used. The protected pentapeptide was cleaved from the resin by treatment of TFA 1% in DCM, filtered and neutralized with pyridine 4% in CH_3_OH. The solvent was removed under reduced pressure and the residue was precipitated in water. The precipitate was filtered and dried. 

*The procedure for the synthesis of deprotected linear disulfide heptapeptide of Longicalycinin A: Cys-Phe-Tyr­-Pro-Phe-Gly-Cys*

A mixture of trifluoroacetic acid / dichloromethane / triisopropylsilane (TFA / DCM / TIPS) (10:10:1) was added to the protected linear disulfide heptapeptide and stirred for one hour. Under such strong acidic condition, side chain- deprotection of the heptapeptide was carried out. Then, the solvent was removed under reduced pressure. The deprotected peptide was precipitated in cold diethylether and collected by filteration.

*The procedure for the synthesis of cyclic disulfide heptapeptide of Longicalycinin A: cyclo-(Cys-Phe-Tyr­-Pro-Phe-Gly-Cys)*

**Scheme 1:**
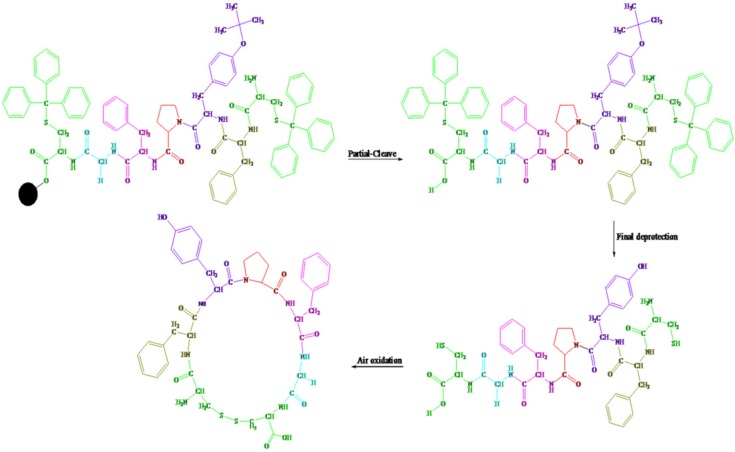
The Solid-Phase Synthesis of linear and cyclic disulfide analogues of Longicalycinin A *via *2-chlorotrityl chloride resin

The deprotected linear peptide (10 mg) was dissolved in water (400 mL) in a round-bottom flask, to which, ammonia (dropwise to reach pH 8-9), as an oxidizing agent, was added and the reaction was aerated under atmospheric oxygen for 8 h. The solvent water was removed under reduced pressure for 1 h at 50 ˚C and the solid was collected (Scheme 1).

*MTT assay*

The synthesized linear and cyclic disulfide analogues of Longicalycinin A were subjected to short term *in-vitro *cytotoxicity study by MTT assay against cell lines of skin fibroblast, HepG2 and HT-29 in 10 μg/mL concentration using 5-fluorouracil (5-FU) as a reference compound. Activity was assessed by determining the percentage inhibition of cell lines. IC_50_ values analogues of Longicalycinin A were calculated using Prism software. At first, the above mentioned cells were cultured in RPMI1640 medium at 37 ˚C under 5% CO_2 _/ 95% air, supplemented with 10% fetal bovine serum (FBS), 100U/mL penicillin and 100µg/mL streptomycin. The cells were seeded into 96-well plates with 10^4 ^cells /well and allowed to grow for 24 h and then incubated with 10 μg/mL concentration of linear and cyclic peptides for 6h. Cell activity was analyzed by using a MTT method which is based on the conversion of 3-(4,­5-dimethylthiazol-2-yl)-2,­5-diphenyltetrazolium bromide (MTT) orange dye to purple formazan crystals by mitochondrial succinate dehydrogenase enzyme in living cells. At the end of each treatment period, MTT (10µL), 5 mg/mL in phosphate-buffered saline) was added to each well and the microplate was incubated at 37 ˚C for 4 h. The medium containing MTT was removed and DMSO (10µL) was added to each well to dissolve the formazan crystals. The plate was incubated for 30 min at 37 ˚C and the absorbance was read at 570 nm using a spectrophotometer plate reader (Infinite® M200, TECAN) (24). 5-Fluorouracil (5-FU) was also used as a positive control and DMSO solvent as the blank for the test compounds. Data are presented as the mean of triplicate measurement of the number of living cells. 

*Flow cytometry analysis*

Flow cytometry analysis was determined by analytical DNA flow cytometry. In this work, HepG2 and HT-29 cells were harvested and adjusted to 10^4^cells/mL and then incubated for 6 h with 10µg/mL concentration of peptide samples. The cells were centrifuged at high speed (12,000 rpm) for 20 s. The pellet was washed with saline buffer, after repeating centrifugation, resuspended in 0.2 mL of lysis buffer (0.1% sodium citrate and 0.1% Triton X-100) containing 50 µg/mL propidium iodide (PI) and stained with this reagent, a highly water-soluble fluorescent compound, at 37 ˚C for 15 min in the dark. The cells were then evaluated for the DNA fragmentation analysis using a FACScalibur flow cytometry instrument (Becton Dickinson, CA, USA) equipped with the flowing software 2.5.1 (25).

*Statistical analysis*

Analyses were performed using Graph Pad Prism 5 (Graph Pad Software, La Jolla, CA, USA). Statistical analysis among groups was performed using multiple comparisons by one ­way ANOVA followed by Tukey’s post hoc test. All data are presented as arithmetic mean ± S.E.M of at least triplicate determinations. Significance was accepted at *P < *0.05.

## Results

*The protected linear disulfide heptapeptide analogue of Longicalycinin A*

*Synthesis of Cys(Trt)-Phe-Tyr(tBu)-Pro-Phe-Gly-Cys(Trt)* (*1a)*

Yield: 54%;Yellow oily liquid; IR (KBr): ν (cm^-1^) 3422.52 (NH amide), 1665.43 (C = O amide), 1546.08 (C = C in amino acids Phenylalanine and Tyrosine), 1194.89 (C-O COOH), 1133.26 (C-O), 600-800 (out of plane bending vibration C-H in amino acids Phenylalanine and Tyrosine); LC-MS (ESI) *m/z* Calcd for (1a) 1375.59, Found *m/z* = 1376.60000(M+1).

*The deprotected linear disulfide heptapeptide analogue of Longicalycinin A*

*Synthesis of Cys-Phe-Tyr-Pro-Phe-Gly-Cys* *(1b)*

Yield: 54%; White solid; IR (KBr): ν (cm^-1^) 3425.87 (NH amide), 1670.51 (C = O amide), 1547.62 (C = C in amino acids Phenylalanine and Tyrosine), 1196.51 (C-O COOH), 1133.44 (C-O), 600-800 (out of plane bending vibration C-H in amino acids Phenylalanine and Tyrosine); LC-MS (ESI) *m/z* Calcd for (1b) 835.31, Found *m/z* = 836.60000(M+1).

*The cyclic disulfide heptapeptide analogue of Longicalycinin A*

*Synthesis of cyclo-(Cys-Phe-Tyr-Pro-Phe-Gly-Cys) (1c)*

Yield: 54%; White solid; IR (KBr): ν (cm^-1^) 3392.49 (OH amino acid Tyrosine), 1726.34 (C = O COOH), 1663.10 (C = O amide), 1346.09 (C = O COOH), 1527-1601.39 (C = C in amino acids Phenylalanine and Tyrosine), 1199.64 (C-O COOH), 1157.97 (C-O), 600-800 (out of plane bending vibration C-H in amino acids Phenylalanine and Tyrosine); LC-MS (ESI) *m/z* Calcd for (1c) 833.31, Found *m/z* = 834.50000(M+1).

*MTT assay results*

The deprotected linear and cyclic disulfide heptapeptides ­of Longicalycinin A have shown cytotoxicity to cancerous cell lines HepG2 and HT-29 in varying degree (ranging IC_50_ values from 10.33 µg/mL to 12.45 µg/mL). Table 1 shows the IC_50_ results for deprotected linear and cyclic disulfide heptapeptides ­of Longicalycinin A along with IC_50 _results for 5-flurouracil, chosen as a standard toxic drug.

*Flow cytometry analysis results*

**Figure 1:**
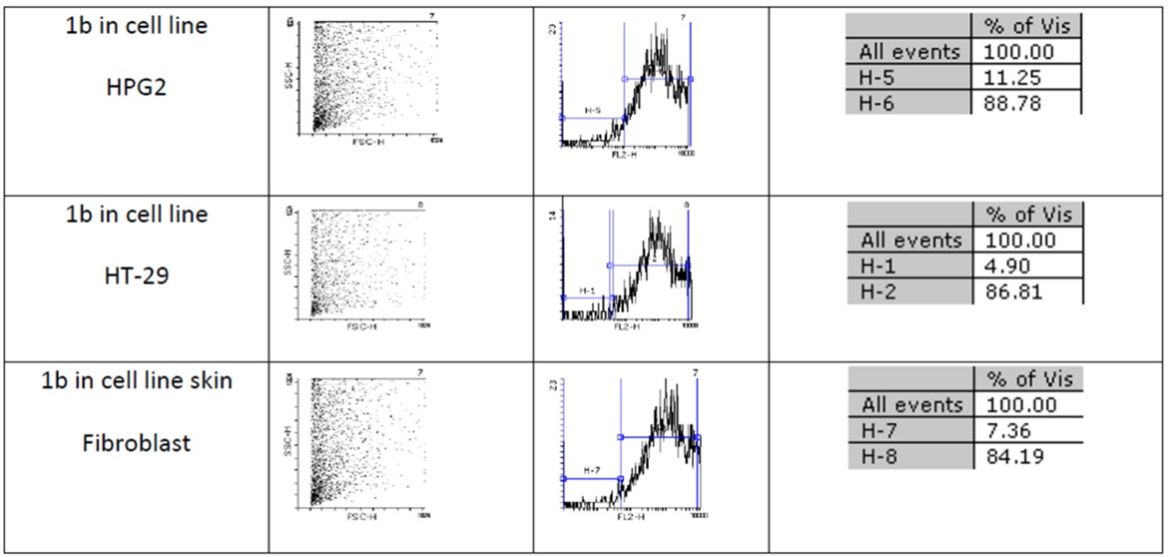
An estimate of the percentage of normal and apoptotic nuclei for 1b by analysis of the DNA histogram after elimination of residual debris.

**Figure 2:**
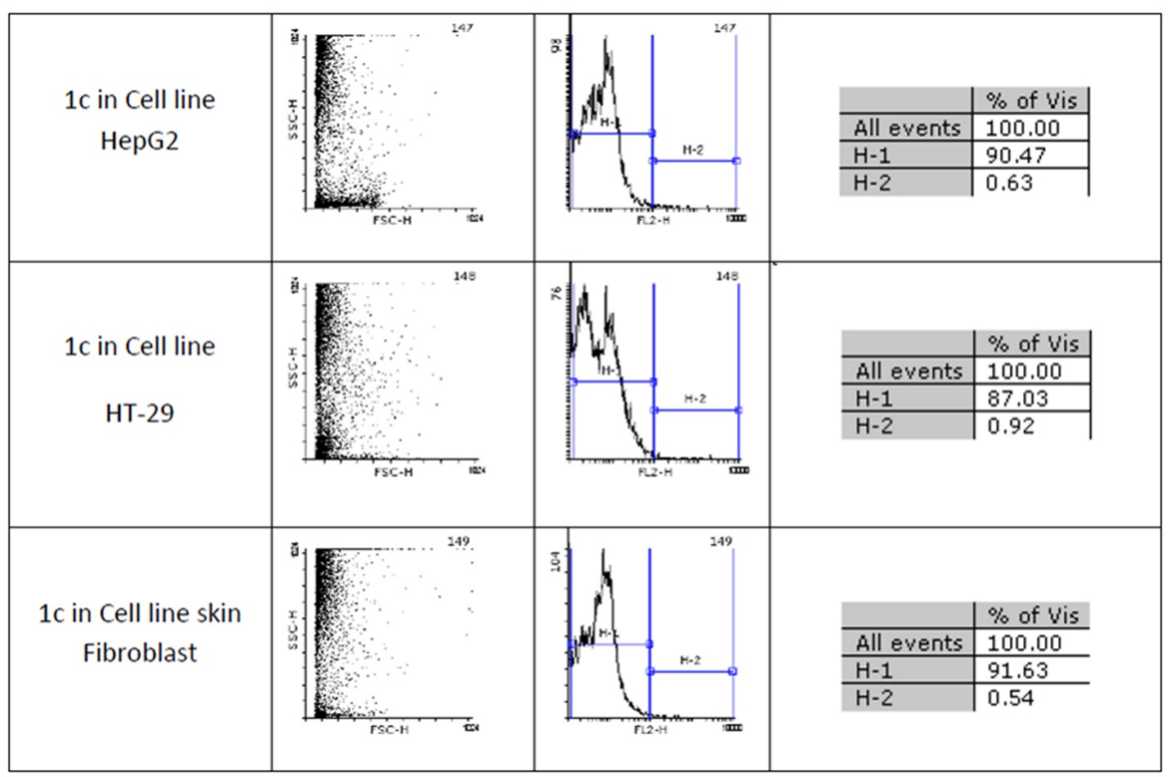
An estimate of the percentage of normal and apoptotic nuclei for 1c by analysis of the DNA histogram after elimination of residual debris.

Figures 1 and 2 show flow cytometery results of linear and cyclic disulfide analogues of Longicalycinin A. The left dot plots present the forward scatter (FSC) parameter in horizontal axis and side scatter (SSC) parameter in vertical axis, which can be correlated with the relative size and granularity of the cells, respectively. The histograms show the intensity of fluorescence of the samples in the FL-2 channel, which corresponds to PI emission wavelength (25)­

## Discussion

Cancer has emerged as the main cause of the highest rate of mortality in the world (26). Drugs used in cancer, although, show some beneficial effects on cancerous organs, demonstrate side effects on normal tissues. On the other hand, anticancer peptides, being effective on target tissues, should be safe, less harmful on healthy organs and can recover the patients rapidly (27). Anticancer peptides with small size (less than 50 amino acids) can penetrate malignant cells easily and be effective through cell-membrane lysing or mitochondria disruption mechanism (28). Both linear and cyclic peptides have shown therapeutic beneficial application in many diseases. Cyclic peptides, compared to linear ones, have an advantage of being orally more stable against lytic enzymes of gastrointestinal tract. The aim of this study was to design and synthesize linear and cyclic disulfide heptapeptides of longicalycinin A that might exhibit anticancer activity more than the original peptide. Furthermore, low toxicity of these peptides on normal cells, if achieved, would be more promising to find new peptide anticancer drug. Meanwhile, structure-activity relationship (SAR) studies of these series, may lead us to reach to the better understanding of molecular basis of cytotoxic activity of these peptide compounds. 

**Table 1:** IC_50_ values (μg/mL) for toxicity activity of peptides towards HepG2 and HT-29 cells. Values are presented as mean ± SD of three independent experiments, performed in triplicate

**Entry**	**IC**_50_ ** cell line HepG2**	**IC**_50_ ** cell line HT-29**
1b	10.37± 0.23 ^***^	12.45± 0.44
1c	10.33± 0.13 ^***^	11.05± 0.42
5-Fluorouracil	3.16± 0.05	6.08± 0.06

*** (*P *<0.001 was considered to be significant).

Two-step synthesis of peptides on CTC resin was chosen as the main strategy in this study due to several advantages achievable from this kind of resin. The trityl linker is readily cleaved under mild acidic condition by treatment of TFA 1% in dichloromethane owing to the high stability of trityl cations (29). Meanwhile desired compound 1b with protected side chains could be obtained in good yield and high purity without facing byproduct as a result of interaction with protection groups releasing in the solution. At the end, deprotection of the side chains of compound 1b was achieved with TFA > 50% containing scavengers. Among the various methods of oxidation, aeration with atmospheric oxygen in a moderate alkaline solution (pH 8-9) was employed in order to cyclize the compound via head to tail disulfide binding (7, 15). Based on our experience such oxidation gave better yield with a higher purity product. Synthesized compounds 1b and 1c exhibited cytotoxic activity against cell lines of HepG2 (human liver cancer cell line) and HT-29 (Human Colorectal Adenocarcinoma Cell Line) with mean IC_50_ values ranging from 10.33 µg/mL to 12.45 µg/mL, comparable with the standard high potent drug 5-fluorouracil (5-FU). The results of MTT assay are shown in Table 1. According to these results, compound 1c with IC_50_ values (10.33 µg/mL) on HepG2 and (11.05 µg/mL) on HT-29 cell lines showed relatively higher toxic activity than synthesized compound 1b with IC_50_ values (10.37 µg/mL) on HepG2 and (12.45 µg/mL) on HT-29 cell lines in Table 1. The histograms of Figures 1 and 2, resulted from flow cytometry analysis of cell lines treated with compounds 1b and 1c, show the intensity of fluorescence of the sample cells in the FL-2 channel. As shown, the histograms of cell lines treated by compound 1b shifted to an upper area (H_2_ area) which refer to remaining normal intact nuclei and the histograms of cell lines treated by compound 1c shifted to a lower area (H_1_ area) which refer to occurring apoptotic nuclei (25). Skin fibroblast cell line as the normal cells with a relative high rate division could provide a safety profile evaluation for the synthesized compounds. From the results of flow cytometry shown in Figures 1 and 2, it can be seen that Compound 1b showed a high safety result on normal cell line (viability percentage 84.19 and apoptosis percentage 7.36) and compound 1c showed a low safety result on normal cell line (viability percentage 0.54 and apoptosis percentage 91.63). Therefore, it can be concluded the cyclic disulfide heptapeptide of Longicalycinin A is more toxic than linear disulfide heptapeptide of Longicalycinin A on cancerous cells as well as on normal cells. 

## Conclusion

Linear and cyclic disulfide heptapeptides of Longicalycinin A showed toxic effects on chosen cancerous cells of liver and colon with varying degrees. Considering the property of toxicity action good enough on cancerous cell lines along with a high safety profile on normal skin cells led us to choose linear disulfide heptapeptide of Longicalycinin A (compound 1b) as a good candidate for further works to make most relevant derivatives to gain structure-activity relationship data and ultimately find active sites for such peptide compounds on cancerous cells. 
